# Positron emission tomography detected thyroid incidentaloma

**DOI:** 10.1308/003588413X13511609956732

**Published:** 2013-01

**Authors:** J Goddard, A Gaunt, DH Markham

**Affiliations:** Warwick Medical School, University of Warwick,UK

**Keywords:** Thyroid surgery, PET/CT, Patient management

## Abstract

We describe the case of a 48-year-old woman who presented with a thyroid lesion incidentally detected on positron emission tomography/computed tomography for a suspicious lung lesion. Subsequent clinical examination and investigations revealed a 3cm nodule in the left lower pole of the thyroid. Fine needle aspiration was indeterminate for malignancy. A left hemithyroidectomy was performed and histology confirmed a benign thyroid adenoma with an incidental micropapillary carcinoma. The literature regarding the best management for thyroid incidentalomas remains uncertain and, as such, each patient must be managed on an individual basis.

## Case history

**Figure 1:**
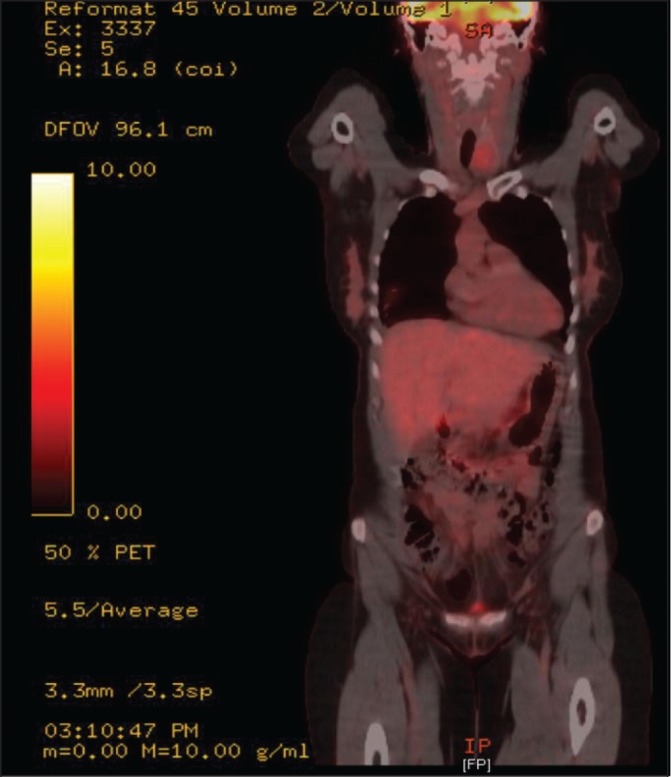
Positron emission tomography showing solitary 3cm left-sided thyroid incidentaloma

In April 2011, after positron emission tomography/computed tomography (CT/PET) to investigate a residual solid right lower lobe lung nodule following pneumonia, a 48-year-old woman was referred to a thyroid surgeon with an incidental thyroid lesion (Fig 1). She had a two-month history of throat discomfort but no voice dysfunction or difficulty breathing. Significant past medical history included right thyroid lobe surgery for a degenerative colloid adenoma 19 years previously. She had never had any thyroid hormone dysfunction and there was no family history of thyroid disease.

On examination, she was clinically euthyroid. There was a visible 3cm nodule in the lower pole of the left lobe of the thyroid. There was no associated lymphadenopathy. Thyroid function tests were normal. Ultrasonography demonstrated a 3.75cm circumscribed, solid, echogenic nodule in the lower pole of the left lobe of the thyroid and radiological appearances suggested a follicular lesion. There was regrowth of the right thyroid remnant. Fine needle aspiration showed follicular cells that were graded as Thy3 (intermediate risk of malignancy).

After discussion with the patient about the alternative management options and in light of the undiagnosed lung lesion, she underwent an urgent left hemithyroidectomy and excision of the right thyroid remnant. There were no post-operative complications.

Pathology of the excised nodule revealed the index lesion was a benign thyroid adenoma. There was an incidental microcarcinoma measuring 0.8mm in the specimen. This was completely encapsulated and not adjacent to the thyroid capsule. The presenting lung lesion was ultimately determined to be unrelated to the thyroid lesion. Resection of the lung nodule revealed an area of necrotising granulomatous inflammation. There were no acid-fast bacilli or fungal bodies seen and there was no clear clinical indication of tuberculosis.

## Discussion

Rates of incidental focal thyroid abnormality on PET have been quoted as 1–2%.^1,2^ A significant number of PET detected thyroid lesions are associated with a palpable lesion on clinical examination.^3^ It could therefore be argued that these are not truly incidentalomas. Truly incidental thyroid lesions have been defined by other authors as clinically impalpable lesions less than 1cm in size.^4^ Our patient had an incidental microcarcinoma within a benign adenoma, which highlights the difficulty of defining the term incidentaloma.

The underlying aetiology of incidental PET detected thyroid abnormalities is not fully reported in the available literature. This is because not all patients with PET detected thyroid abnormalities have a histological diagnosis. This is for a number of reasons. Primarily, PET is most commonly undertaken for staging of known non-thyroid malignancy. Patients are therefore often not fit enough to undergo further investigations that will not affect their prognosis.^3^ As our patient had an undiagnosed lung lesion, no known underlying malignancy, and was generally fit and well, she was investigated.

The PET positive thyroid abnormalities that are associated with an increased risk of thyroid malignancy include focal lesions, unilateral involvement and a palpable nodule on clinical examination.^2,3^ This patient displayed all of these features.

## Conclusions

The literature on the nomenclature of incidentalomas, their aetiology and the exact risks of malignancy remains unclear. The best management strategy for patients therefore also remains uncertain. The stress and anxiety that additional appointments, investigations and diagnostic uncertainty will cause individual patients at a time when they are already undergoing investigations for possible malignancy cannot be underestimated. Ultimately, each patient needs to be dealt with on an individual basis owing to the likely complexity of other ongoing medical investigations and diagnoses.
